# “They seemed to be like cogs working in different directions”: a longitudinal qualitative study on Long COVID healthcare services in the United Kingdom from a person-centred lens

**DOI:** 10.1186/s12913-024-10891-7

**Published:** 2024-04-01

**Authors:** Chao Fang, Sarah Akhtar Baz, Laura Sheard, J. D. Carpentieri

**Affiliations:** 1 Department of Sociology, Social Policy and Criminology, University of Liverpool, Liverpool, UK; 2 Department of Health Sciences, University of York, York, UK; 3 Department of Education, Practice and Society, Institute of Education, University College London, London, UK

**Keywords:** Long COVID, Healthcare, Chronic illness, Person-centred care, Health services, Longitudinal, Qualitative

## Abstract

**Background:**

The COVID-19 pandemic has presented significant challenges to the already over-stretched healthcare system in the United Kingdom (UK). These challenges are particularly pronounced for people living with the novel condition of Long COVID (LC) as they often face persistent and fluctuating symptoms, encountering prolonged uncertainty when seeking medical support. Despite a growing understanding of the healthcare challenges associated with LC, existing qualitative studies have predominantly focused on individual experiences rather than examining the structural aspects of healthcare.

**Methods:**

A longitudinal qualitative study with 80 participants and 12 healthcare practitioners was conducted in the UK to explore the healthcare experiences of those with LC. In total, 178 interviews (with attrition) were collected across two rounds, from November 2021 to March 2022, and from June to October 2022.

**Results:**

Embracing a person-centred framework that recognises and nurtures interconnected individual, relational, and existential needs, we investigated healthcare experiences related to LC across primary, secondary, and specialist integrated care. Using this perspective, we identified three overarching themes. Theme 1 addresses the persistent hurdle of accessing primary care as the initial point of contact for LC healthcare; Theme 2 underscores the complexity of navigating secondary care; and Theme 3 encapsulates the distinctive challenges of developing LC integrated care. These themes are interlinked, as people with LC often had to navigate or struggle between the various systems, with practitioners seeking to collaborate across the breadth of their professional responsibilities.

**Conclusion:**

From a person-centred approach, we were able to identify the needs of those affected by lasting LC symptoms and comprehend how health services intricately influence these needs. The focus on healthcare systems also captures the nuanced impact that continuing healthcare struggles can have on people’s identity. As such, our findings provide evidence to inform a more effective and sustainable delivery of person-centred care for people with LC across various healthcare settings and over time.

**Supplementary Information:**

The online version contains supplementary material available at 10.1186/s12913-024-10891-7.

## Background

The COVID-19 pandemic has presented significant challenges to healthcare in the United Kingdom (UK). At the peak of the pandemic in 2020–21, the Office for National Statistics (ONS) estimated that over 10% of the UK population suffered from persistent symptoms 12 weeks post-infection [1]. According to the latest ONS statistics from March 2023, around 1.9 million people (2.9% of the UK population) self-reported Long COVID (LC) symptoms [2]. These symptoms can encompass respiratory, cognitive and cardiovascular issues. Commonly reported issues include fatigue, breathlessness, brain fog and pain, while others also encounter hair loss, skin rashes and sensory dysfunction [3]. Managing LC symptoms is often closely connected to its multisystemic and often changing nature, requiring ongoing support for symptom management and recovery [4]. While understandings of and treatments for LC as a new illness are fast-evolving, healthcare provision in the UK continues to lag [5]. Extensive literature has painted a troubling picture of fragmented healthcare for LC: people living with LC (PLwLC) often have to do the “hard and heavy work of managing symptoms and accessing care” [6] and thus face various challenges arising from their disrupted health and identity [7, 8].

The UK healthcare system has been scrutinised by waves of PLwLC across different levels [2]. A scoping review on potential LC pathways [9] suggests that LC related healthcare has been predominantly offered in primary care, yet accessing it remains a significant hurdle for many [10, 11]. Even when admitted into healthcare, they also faced considerable barriers in navigating said systems to obtain more specialised (often secondary) care for their symptoms [4, 7]. To meet the unique needs of PLwLC and to optimise healthcare resources dedicated care pathways have been established across the UK since December 2020 [12]. These pathways, including Long COVID clinics in England and similar services in Wales, Scotland, and Northern Ireland, aim to offer tailored care that addresses the multifaceted challenges (e.g., physical, mental and social) individuals face during their LC trajectories. However, these pathways are limited due to lacking accessibility and holistic care [13]. The dilemmas between the needs of PLwLC’s and the constraints of healthcare provision have reflected a “perfect storm” in a “healthcare system which has faced years of austerity, budget caps, increasing waiting times, pressurised services, backlogs, and workface shortages” [14].

These healthcare dilemmas for LC have so far been captured *as experienced by individuals*. Drawing upon individual narratives (e.g., both the general public living with PLwLC [4, 6, 7] and healthcare professionals with LC [4, 15]), current literature has often explored healthcare as a vague (broad) construct, asserting that people with LC were “let down, fought against and negotiated with” [6, 8, 10]. In other words, LC healthcare has been primarily examined through subjective feelings and the experiences of individuals. Various theoretical frames have been used to explore both the complexity of dilemmas facing LC healthcare and the potential solutions for these. Researchers have employed lenses such as “candidacy” [16], “legitimation” [17], “epistemic injustice” [8] and “sick role” [18] to capture both the challenges and the agency of PLwLC’s interactions with healthcare. While the focus on individual encounters in healthcare is valuable, from a health service research perspective, it is also important to understand barriers and enablers for LC healthcare from a more systematic and structural perspective. As such, these understandings can contribute to more explicitly identifying gaps in care provision, offering evidence to further shape healthcare development for LC in the longer term.

To enhance the understandings of PLwLC’ needs and how these are supported, undermined and overlooked in healthcare systems, we argue that adopting a person-centred lens is valuable. Person-centredness, as both a theoretical and practical framework, has long been a cornerstone of healthcare. It prioritises the individual needs and values of patients throughout their healthcare journey. Research has suggested that developing healthcare provision with a person-centred lens can contribute to improved health outcomes and reduced care expenditure [19, 20]. This is particularly important in the UK as the healthcare services are free at the point of delivery and the National Health Service (NHS) is responsible for providing comprehensive healthcare to the whole population across a range of interconnected care settings, including primary, specialist, acute/emergency care and integrated care pathways [21].

In the context of this universal healthcare, the values of person-centred approaches have been interpreted and implemented in a multitude of ways, aiming to treat patients as a person whose ‘‘choice" and "rights" are holistically and consistently addressed [20]. This focus of person-centred care also entails an empowering dimension, ensuring patients are equipped with sufficient knowledge and skills, and are confident in managing their own health [19, 22]. Healthcare in the UK thus far has actively sought to promote patients’ individual wishes, dignity and autonomy across all the processes of care planning, treatment and decision-making [23]. Person-centred needs have also been closely examined in the rich matrix of patients’ family, social, cultural and religious circumstances to support their relational being [20, 22]. Meanwhile, there is also an increasing focus on patients’ existential needs in healthcare settings particularly for those with terminal and chronic illnesses, in response to deeper fears of losing fundamental resilience and meaning due to compounded health-related losses [24]. More importantly, these individual, relational and existential components of person-centred care are often emphasised not as separated but interconnected entities in healthcare provision [21, 23]. As such, person-centred care presents a holistic and systemic approach to addressing the multifaceted and interconnected needs of people with health conditions throughout their healthcare experiences [24].

Despite efforts to integrate person-centred care into the UK healthcare system, persistent gaps, primarily attributed to funding and staff shortages, hinder its implementation, leading to increased waiting times and reliance on self-management or private healthcare. Regional disparities, inequalities, and factors such as racism, age, gender, and cultural differences further obstruct healthcare engagement with patients' multifaceted needs. These challenges to the implementation of person-centred healthcare in the UK have been further exacerbated by the COVID-19 pandemic [5]. The unprecedented crisis has heavily drained the already over-stretched public health resources, amplifying existing issues such as staff shortages, data sharing, inter-organisational coordination and inequalities [14]. As a result, enacting person-centred care may become even more complicated in varied healthcare settings [25]. The COVID-19 pandemic has also presented unique circumstances, confronting the competing agendas of prioritising individual care and public health interests. As such, person-centred care is a useful lens to explore how individual needs are shaped by standardised (and highly pressurised) healthcare structures. This lens can be particularly beneficial for critically understanding healthcare services for people with LC, as living with such novel and persistent symptoms may require both acute care and prolonged support alongside their illness trajectories. Ultimately, we aim to understand not only the quality of health care provision but also to more adequately clarify how LC patients’ needs are supported/undermined in (intersection with) health systems over time. These understandings will help to inform the continuing development of holistic healthcare for LC and other similar chronic or novel health conditions in the UK and internationally.

## Methods

We conducted a longitudinal qualitative study to understand the evolving needs of PLwLC in the UK and the evolution of healthcare in response to this. The interviews were conducted at two times between 2021–2022, respectively November 2021–March 2022, June–October 2022 (each participant had their two interviews approximately 6 months apart). The findings of the first round of interviews have been previously published [10, 31].

### Research design

Our study was designed to conduct three phases of interviews over 2021–2023, talking to both people living with LC and healthcare practitioners. This article reports upon our findings from the initial two phases which extensively explored healthcare experiences (the third phase, conducted in early 2023, focused on resilience). Our aim in this study was to capture an ongoing picture of LC experiences and healthcare across the UK. We also placed a specific focus on a region in northern England characterised by great levels of deprivation (to as region A to project the confidentiality of our participants). This geographical focus allowed us to further contest social and racial inequalities in healthcare, which were exacerbated during the COVID-19 pandemic [26]. Our investigation examined experiences and development of LC health services from both cross-sectional and longitudinal perspectives. Cross-sectionally, by combining data from both PLwLC as (potential) health services users and healthcare professionals as services providers, we aimed to gain a fuller understanding of how people with LC are (or not) supported as a ‘person’ in health services at that time [19]. Longitudinally, we then tracked how person-centred healthcare for LC was developed over time in the fast-evolving context of LC as a social and medical experience.

### Sampling

To better understand provision and experience of LC healthcare across diverse circumstances, we used purposeful sampling to recruit participants with self-identified LC from nationally and regionally representative cohort studies. This approach enabled us to not rely on health records for recruitment, as such not only reaching those within healthcare systems (and thus on health records) but also PLwLC who have not accessed healthcare for a variety of reasons. Focusing on self-identified LC symptoms provided us access to people who have not been formally diagnosed with COVID and LC, whose voices often remain unheard due to early testing limitations and healthcare access issues. Our sampling strategy also expanded our research scope by including PLwLC who may not have a strong online presence or digital literacy, unlike many earlier studies recruiting from self-selecting online groups [7, 16]. This inclusive sampling strategy allowed us to engage with people residing in diverse, often overlooked, social and community settings to explore their healthcare experiences of living with LC [10].

**Fig. 1:**
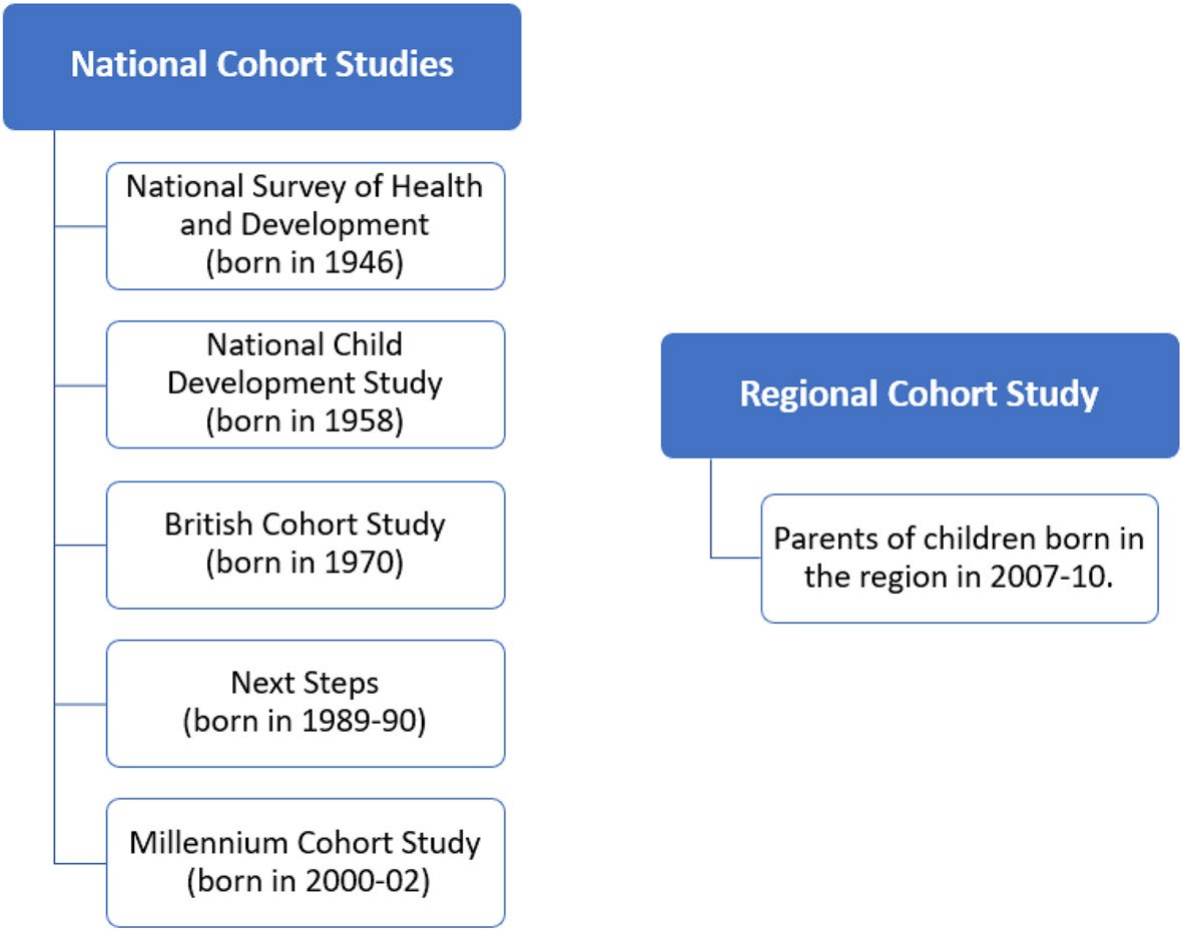
Participating cohort studies

Participants living with LC were recruited in two ways. Firstly, 40 participants were recruited from five UK national cohort studies (Fig. 1). Specific COVID-19 surveys from these cohort studies identified cohort members who reported COVID-19 related symptoms for over 4 weeks, with a focus on those indicating symptoms for over 8 weeks. Recruitment concluded upon reaching the target of 40 interviews. Secondly, 40 interviews were collected in Region A. A similar recruitment approach was used (Fig. 1**)**, identifying 21 participants, who are parents of children born in a general hospital in Region A between March 2007 and December 2010. The remaining 19 participants were recruited from the wider local community through community workers and snowball sampling, to gain a more diverse demographic sample. Both our national and region-specific recruitment oversampled individuals with greater socioeconomic deprivation and from ethnic minorities to reach people who may be underrepresented in other LC studies. In the first phase of interview collection, we conducted in-depth interviews with 80 socially and ethnically diverse participants across varied age groups, including two participants in the 70–79 age group and their partner in a dyad interview (Table 1). 73 participants, including the two with their partner, remained for our second phase of interviews (9% attrition rate). Over the two phases, around 25% of PLwLC recovered, 25% improved but had minor symptoms, and the remaining half continued to experience significant and sometimes worsening symptoms (these are estimates due to some participants that their symptoms intersected with other health conditions).

**Table 1:** Descriptive information about participants living with LC

Information on participants living with LC over two phases of interviews		
	***Sample(N =80)***	***Sample(N =73)***
	**Phase 1**	**Phase 2**
**Gender**		
Female	56	54
Male	24	19
**Age**		
18–19	2	1
20–29	6	6
30–39	20	18
40–49	18	16
50–59	15	15
60–69	14	13
70–79	5	4
**Ethnicity**		
White British	43	40
South Asian	30	27
Southeast Asian	2	2
White other	3	3
Mixed	2	1

To enrich our picture of LC health services, particularly from the perspective of providers, we included healthcare and public health professionals in our study. We adopted an inclusive approach, aiming to engage interdisciplinary professionals from different healthcare sections supporting LC (e.g., primary and specialist care). We concentrated on sampling practitioners in the metropolitan region A, including the city and surrounding areas. This strategy allowed us to explore how inequalities, such as poverty and racism, may disproportionally contribute to the healthcare challenges facing PLwLC. We also paid attention to the provision of the LC pathway in Region A, one of longest-running pathways in the UK [27], particularly within the limited timeframe of our study. Using our team’s existing connections as well as snowballing, 12 practitioners were recruited from 7 different care settings in the first phase of interviews (Table 2). In phase 2, we talked to 13 healthcare professionals, including 9 remaining from the first phase and 4 new recruits due to job changes and the development of new services.

**Table 2:** **Descriptive information about healthcare practitioners supporting people living with LC**)

Information on practitioners over two rounds of interviews			
		***Sample(N =12)***	***Sample(N =13)***
		**Phase 1**	**Phase 2**
**Health systems** **(region A)**	**Profession**		
Primary care	General practitioner	2	2
Secondary/LC specialist care	Service/project manager	2	1
	Lead clinical practitioner/coordinator	3	6
	Physiotherapists	2	2
	Occupational therapist	1	1
Community care	Head of a charity working with community health and social services	1	1
	Local council public health official	1	0

All the interviews with PLwLC and practitioners were conducted remotely, either online by Zoom or telephone (apart from with two PLwLC who had hearing accessibility issues). The interviews ranged from 30 to 110 min in length and averaged around 45 min.

### Data analysis

We adopted a reflexive thematic analysis approach to interpret the rich data [10]. Given the longitudinal nature of our study, the data analysis presented in this paper focused on "changes" in healthcare both from an individual and structural perspective (by talking to both PLwLC and practitioners). As such, we “ground the interviews in an exploration of processes and changes which look both backwards and forwards in time [28](p.194)”, to explore the construction of person-centred healthcare in response to the novel and often complex condition of LC in the UK. To gain in-depth and critical insights into LC healthcare, we analysed the longitudinal qualitative data at three levels, namely, “description”, “analysis” and “interpretation” [29]. These levels aimed to understand “if/what changes had occurred”, “how/why these changes might have happened” and “what is the meaning and impact of these changes”.

**Fig. 2:**
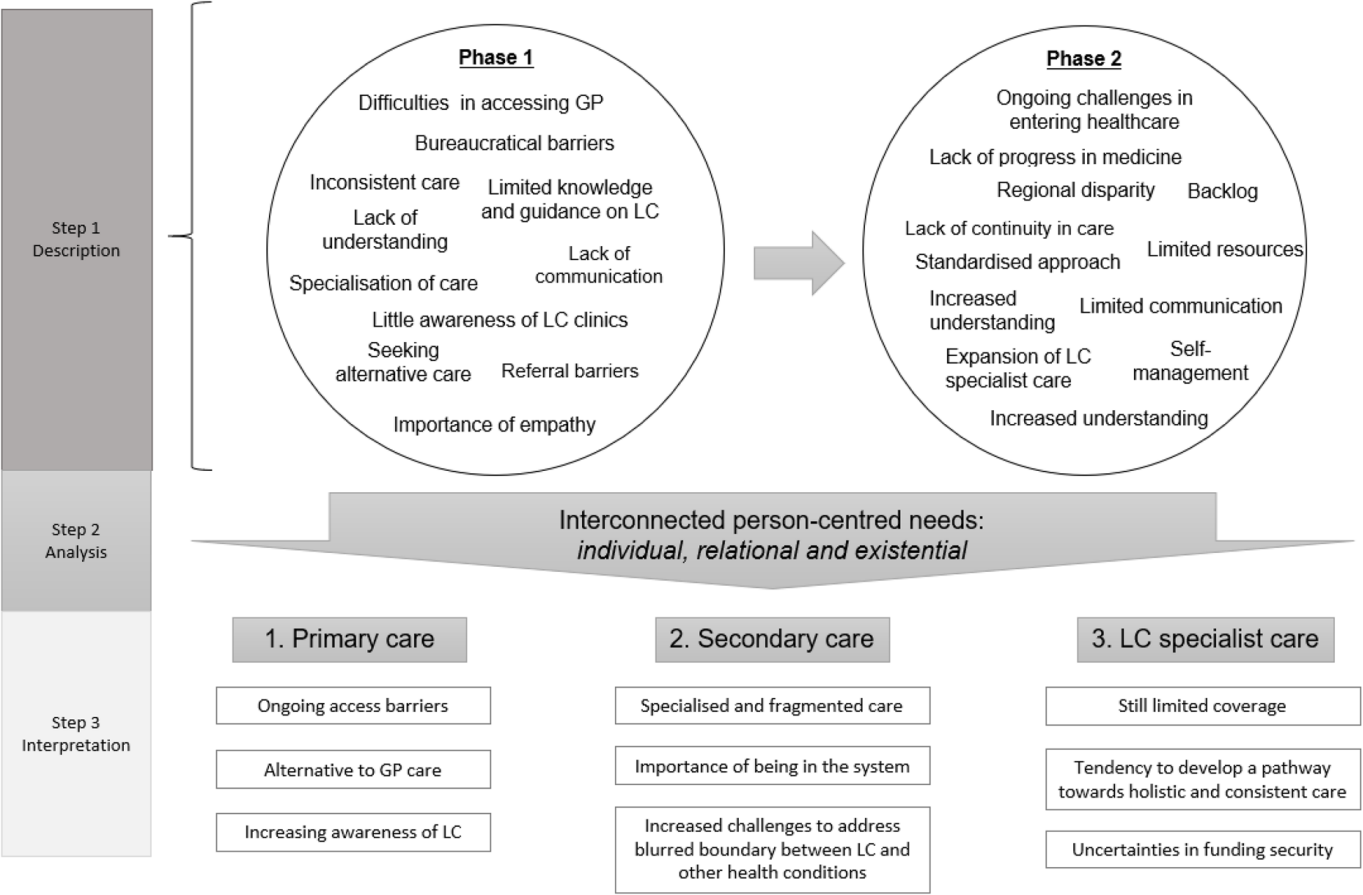
An illustrate of our coding process

Following these guiding questions, we conducted a three-step analysis (Fig. 2). Firstly, we generated and compared initial codes from both phases to describe the provision and development of LC healthcare over time. Based on step 1, we moved to step 2, incorporating a person-centred lens [24] to analyse essential features of these developments and the systematic interconnections amongst these changes. Finally, in step 3 we interpreted how these changes address/overlook PLwLC as a ‘person’ from a holistic and consistent perspective. Throughout the analysis, we considered changes both at the individual and the collective levels to more fully capture changes in LC healthcare and the impact on individual experiences. During this threefold longitudinal analysis, we also carefully relate, contrast and further incorporate narratives from both PLwLC (service users) and practitioners (service providers) to explore the intersections between broader healthcare structures (e.g., operation, implementation and care delivery) and the localised experiences of PLwLC’s multifaceted and persistent needs.

## Findings

We present our findings on LC healthcare services in the UK broadly through three dimensions of the health system: primary, secondary and LC specialist care. While acknowledging that services also exist between and outside these systems, we briefly mention them to provide a broader view of LC healthcare. Figure 2 shows three overarching themes: theme 1 reports continued hurdles in accessing the first point of contact for LC healthcare; theme 2 highlights the complexity in progressing through secondary care; theme 3 captures unique challenges to the propagation of integrated LC care pathway. These themes are used to capture a systemic view of how people with LC are (or are not) supported in a person-centred manner. Our findings and the three dimensions of healthcare they encompass are interconnected. As we found, people were often bounced between these systems; practitioners also sought to cooperate across their own remit with practitioners from diverse fields.

The person-centred lens here is used as an overarching framework providing a consistent thread to understand the quality of healthcare for PLwLC. This lens focuses on multifaceted needs, including individual, relational, and existential aspects, all of which are interconnected, shaping their health experiences and identity in nuanced ways. As such, we examined how these needs are address/overlooked both within specific and across healthcare systems, exploring how person-centred lens can inform healthcare services, to more holistically (multifaceted needs) and consistently (across settings and time), to support LC. To protect participants’ confidentiality, pseudonyms are used throughout below.

### Theme 1: continued hurdles to accessing primary care and to entering healthcare systems

#### Ongoing access barriers

Primary care as the first point of contact with healthcare is often the principal gateway for people to enter health systems [30]. A predominant message voiced by our participants across the two phases of interviews is the significant barriers in accessing primary care, particularly general practice (GP) services, including not getting an appointment, receiving inadequate care and having to be persistent. Research has found that the COVID-19 pandemic has created a “perfect storm” to exacerbate the already pressurised primary care services [10, 15].

During the first phase of interviews in 2021–22, over 70% of our 80 LC participants faced significant challenges in securing GP appointments for their acute and persistent COVID-19 symptoms. Reported access barriers included “waiting on the phone for over two hours”, “making 100 calls on a Monday morning”, as well as “being turned away by receptionists or nurses before seeing a GP”. The struggle with access is vividly captured by Barry’s experience:

Barry: “*You’re almost like a leper going to the GP, just can’t get to the surgery… I just can’t see a doctor. It’s all done like this. You know, sometimes just done over the phone, it’s not even a video.*” (Male, White British, 60s, phase 1)

The situation had not improved significantly later in 2022, the rate of participants in phase 2 experiencing GP access barriers remained high, with over 65% affected. This continued delay and inaccessibility was also acknowledged by the GPs who we interviewed in both phases, reflecting long-standing issues related to budget caps, backlogs and workforce shortages in primary care. The ongoing hurdles in obtaining basic medical support, as well as clarification and reassurance, led to increased frustration and health-related anxieties among many of our LC participant:

Susan: “*You don’t know the fact that maybe you haven’t had treatment or things haven’t been picked up, maybe will impact your long-term health because you’ve been waiting for so long. I think that’s also the thing, have I missed the time to get some treatment or something?*”. (Female, White British, 50s,)

#### Alternative to GP care

Due to the ongoing barriers to accessing primary care, many LC participants had trouble accessing NHS healthcare for medical support, leading them to explore alternative ways to address their health concerns. They often resorted to self-management, using methods like painkillers, supplements, exercise, and meditation to cope with their symptoms. Some sought advice for LC symptoms from resources like the Internet (particularly online self-help groups) and local communities, including pharmacists. Only a small number of participants (just seven in phase 1 and five in phase 2 interviews, respectively) were able to access private GP services.

Despite ongoing barriers to accessing primary care, some participants, particularly those with urgent symptoms, turned to emergency care for faster and more immediate access to healthcare. However, emergency care often provided one-off support without follow-up, leaving participants like Sara without continuous assistance for their ongoing struggles:

Sara: “*I went to hospital and had x-ray, blood tests done and they said ‘you’ve got a blood clot, we think it’s on your lung but we need to book you for a CT scan’… [following the scan] I came home and they gave me some blood thinning tablets and after about 10 days I had a CT scan. Whatever it was the blood clot was not there anymore. They said that ‘there’s some damage on the lungs’. So they gave me some inhalers and I got to go home. After that I wasn’t well at all… They could clearly see that but what the long effect is we don’t know. But I’ve not been back to the GP to find out, the only follow-up appointment I was offered was in hospital with the blood clot.”* (Female, British Pakistani, 30s, phase 1)

In addition, community-based care, such as physiotherapy, was another avenue for alternative care; however, access to it often had to be gained through primary care. When asked about how physiotherapists identify people with needs related to LC symptoms, Luke, who was a PLwLC and a volunteer in a local therapy centre, said (this issue remained in phase 2 interviews):

Luke*: “That’s what we’re waiting on the hospital to do for us. And we haven’t had any referrals yet. I don’t know what the hold-up is. We’ve not, we are all geared up ready for them, but we haven’t had anybody come through.”* (Male, White British, 60s, phase 1)

The above experiences underscore the ongoing call to emphasise the significant role of primary care in LC healthcare [9, 30]. Nonetheless, due to persistent access barriers and/or previous negative healthcare experiences, approximately half of our participants felt “exhausted” and had to “give up” trying to engage with their GP and enter the healthcare system. The number remained persistent in phase 2, further fuelling people’s anxiety and worries about their symptoms.

#### Increasing awareness of LC

Despite a rather disheartening picture depicted above, we also observed improvements in primary care for PLwLC during our two-phase interviews (November 2021 – October 2022). In phase 2, some of our LC participants reported increased awareness of LC as a novel medical condition among professionals. These improvements in primary care were also supported by accounts from primary care practitioners, with one GP mentioning that she had more information to draw upon to recognise and empathise with patients’ LC symptoms:

“*I’ve seen patterns I suppose of what people have had. So certainly right at the beginning, it was all the lost your taste and smell and you’re going to have a cough and breathlessness, particularly kind of neuro type symptoms. Those kind of things I can reassure patients that I’ve seen and that people generally tend to get better, so you get a bit more idea of what kind of symptoms are coming.” (GP 1, phase 2)*

While some patients experienced improved support, or at least understanding from their GP, this increased awareness of their condition was not regionally/nationally consistent. According to a GP we interviewed, this discrepancy may be attributed to the lack of “systemic training and knowledge” about LC, especially during the early days of the pandemic, for primary care practitioners. In other words, whether and how to support LC was often dependent on practitioners’ own wishes/opinions/understandings. As a result, a contrasting picture was observed, with a few patients reporting improved primary care while many others still found the improvement limited. Craig shared that although his GP acknowledged his LC symptoms, she tended to be "reactive" rather than "proactive" in providing support.

Craig: “*She took note of it [LC], obviously, we’d had it– but didn’t prescribe or didn’t expand on the conversation. She was just more reactive as opposed to proactive on it.”* (Male, White British, 50s, phase 2)

This ongoing inadequacy and disparities in LC support from primary care may contribute to the exacerbation of people’s worries and even desperation. During our phase 2 interviews, some participants expressed the sentiment that “there is nothing they [GPs] can do”, with a few opting to forgo primary care in favour of alternative forms of support.

As illustrated in the theme above*,* we identified barriers in LC primary care, termed "de-personalisation" by one PLwLC participant. While this phenomenon was not unique to primary care and was also observed in secondary and specialist care settings, it was particularly significant as it created a sense of neglect at the gateway to the health system. Many participants with prolonged and often 'mysterious' LC symptoms faced various hurdles in accessing primary care for initial clarification and support. Even those, who gained access, often struggled with limited understanding and support from practitioners as the first contact point with professional healthcare. This could further illegitimatise their experiences and failed to validate their suffering. Despite ongoing efforts to improve LC support in primary care, the continued uncertainties around the condition seemingly caused significant emotional distress, including frustration, anxiety and fears, related to both healthcare access and their illness. Participants often likened the differentiated progress in primary care to a "postcode lottery", adding to the uncertainty they felt about their healthcare.

Failing to meet the individual and relational needs of PLwLC in primary care may have further implications for their existential needs [31]. These continued multifaceted uncertainties may hinder their ability to access clinical and social support to understand and adapt to this novel health condition. These experiences can be particularly existential, as exemplified by Susan's concerns about her shattered health and future due to limited healthcare access. Without sufficient clarification and reassurance from reliable medical resources, PLwLC may struggle to retain meaning and purpose as they navigate their ongoing life with the illness [31].

### Theme 2: complexity in navigating secondary care

#### Specialised but fragmented care

Secondary care, compared to primary care, tended to provide more specialised support for many of the often-complicated symptoms faced by PLwLC. However, due to the limited availability of secondary care and the ongoing issues around access to primary care, only 17 out of 80 participants had access to secondary care in phase 1 (e.g., for cardiological, respiratory, neurological and mental health complications). In phase 2, the situation remained largely similar, with 14 participants being discharged due to their improved conditions while 5 more with worsened symptoms were being referred to secondary care. While these participants appreciated the targeted care they received, they also felt that the highly specialised nature of secondary care sometimes could restrict their chance to access more holistic and integrated care. Lucy found her fatigue and brain fog symptoms were not adequately addressed by varied specialists:

Lucy: “*The thing I found the most difficult about the healthcare system is that they don’t see things as a round so that you know you kind of go and see a few different specialists and one’s looking at your lungs, and one’s looking at your head, and one’s looking at your heart but nobody’s pulling all together and sitting down and going, this is what we think. There’s no, you’re not really sure what the journey is or what they’re trying to find out, what the conclusion is.”* (Female, White British, 50s, phase 1)

The above situation was not particularly better in phase 2 as Lucy and her peers still struggled to access what she described as ‘holistic care’. This over-specialisation could also be attributed to a lack of communication across healthcare settings. A GP reported her frustration, saying, “*I want to know as a GP that my patients have been worked up for properly. I have to ask secondary care [for updates] because it’s out of my hands”* (phase 1 interview).

The inconsistency within and beyond secondary care for PLwLC could also cause frustration. In our study, only a few participants, such as Heather, who had a certain level of healthcare knowledge and communication skills, could navigate the highly specialised and fragmented care systems, often employing extreme persistence at the cost of becoming known as “infamous”:

Heather: “*I phoned [hospital A] the next day because although it's another hospital called [hospital B] in [location C], it was through [hospital A] that I had to try and weave my way through. And thankfully, I'd taken so many phone numbers when my husband was at [hospital A], I managed to go through to a specialist and say, ‘I can't get through to your booking clerk, but I need to do this’, and because we're infamous now I think, [laughs], I managed to get – she got me an emergency appointment for the afternoon.*” (Female, White British, 60s, phase 1)

Heather’s situation in phase 2 was “slightly better but not significantly”. After enduring prolonged struggles while navigating secondary care and beyond, Heather expressed that “*they [healthcare professionals, such as specialists, paramedics and receptionists] seemed to be like cogs working in different directions*”. This disintegration of care provision was not unexpected to our participants working in secondary care. A respiratory consultant highlighted that "*this is not just a problem for Long COVID, but a broader issue*" due to a lack of funding and staff (phase 2).

#### Importance of being in the system

Despite the barriers to accessing holistic and consistent secondary care, our LC participants predominately emphasised the importance of being in the healthcare system (particularly secondary care after GP referrals). Those referred/admitted to secondary care (e.g., hospitalisation, specialist care), often had positive healthcare experiences. For instance, Patrick entered secondary care via hospitalisation for his acute COVID-19 symptoms. Whilst he had to wait for and navigate various specialist care pathways over a lengthy period after hospital discharge, he still found the support he received “helpful” for his LC recovery:

Patrick: “*They [the hospital] were very supportive – after I left hospital there was in total about seven or eight months of follow-up support. I had the general nurse, the general consultant because they were worried about strokes and they were worried about different things, neurosurgeon, a lot of different people doing bloods. So I had to have the different people signed off and occupational psychiatry had to sign me off etc. So bit by bit they all signed me off until I got out and then even thereafter each function needed to monitor you afterwards. So I had a lung specialist because my lung was damaged. I had a haematologist, so different functions… Definitely, I mean the support helped me recover*.” (Male, British Indian, 50s, phase 1).

Such support in secondary care was afforded by concentrated resources and the highly specialised nature of treatment, ensuring patients are “healthy enough” to be discharged and referred back to primary care [9]. Therefore, many of our participants were determined to enter the specialised secondary care system. This struggle was particularly evident in Gemma’s persistence, expressed across the two interviews, as she wanted her “*Long COVID to be logged in the NHS*” for referral and further examinations/treatment of her multisystemic symptoms of fatigue, pain and sensory issues (Female, white British, 63).

These attempts to enter and move forward through the system however were often obstructed by barriers in secondary care, and sometimes connected to primary care, reflecting broader issues in the UK public healthcare system. A service manager reported ongoing efforts to clear referral delays at her hospital over the two interviews, highlighting both the healthcare access barriers and the impact on the wellbeing of PLwLC:

“*Sometimes there are delays in getting the bloods and things like that, I think there is a huge barrier to people even getting an appointment with their GP to get referred and that can lead to a lot of helplessness and hopelessness for people like, what’s the point and will I get any help anyway? That’s a huge barrier, I hope that people know that widely in society what’s available and I think the challenge is each local commissioning area have a different pathway*.” (Service manager 1, phase 2)

#### Increased challenges to address blurred boundaries between LC and other health conditions

Secondary care faces ongoing challenges due to the novel and rapidly evolving symptoms of LC. Our conversations with participants during 2021–22 revealed that LC symptoms often develop alongside other pre-existing health issues, creating complications that can be challenging to address even with highly specialised care. Some interviewees expressed frustration with the lack of adequate support available to manage their LC symptoms alongside other health concerns. For example, Linda's hospital admission, reported in her phase 1 interview, was unable to determine whether her throat problem was related to LC or another underlying condition. In the second interview, she continued to express her concerns about changing LC symptoms and her desire for more specialised/personalised healthcare.

Linda: “*Obviously, symptoms are still coming up and changing, so it’s not that you’re living with long term symptoms that are the same, that actually symptoms are changing, and new symptoms are coming along, that weren’t there a year ago; which is very odd… That’s why you want to get to be seen [by specialists], so if they start to see something worrying coming out, that you’re on a list somewhere. At the moment I don’t feel I’m on a list anywhere of people that are suffering from this*.” (Female, white British, 30s, phase 2).

The above accounts highlight the importance of healthcare providers addressing the interconnected nature of LC symptoms and other health issues for more effective care. For instance, in a phase 2 interview with a hospital service manager, a respiratory care service shifted to acknowledge that the symptoms of middle aged PLwLC could potentially be compounded by LC and menopause:

“*We’re realise that women who are of menopausal age those have been affected more by post COVID as well. We’ve done some training sessions on the menopause and post COVID to try and again make people more aware.”* (Service manager 2, phase 2)

Despite the positive developments observed in region A, the majority of participating PLwLC faced persistent barriers in accessing specialist care for their increasingly interwoven symptoms between LC and other conditions. In some cases, due to the specialised nature of secondary care, PLwLC with complex and unknown symptoms could face exclusion from the healthcare system. Penny was one of those who was already ‘in the system’ due to pre-existing health issues, but had to re-enter specialist care via the aforementioned challenging primary care routes for her LC-related pains that intersected with her previous conditions:

Penny: “*Like my rheumatoid I know when I’m having a flare up, I visit the consultant and I have a good back-up with the rheumatoid team. If I’m having a flare up or something’s wrong, I know that I phone up, the rheumatoid team and they help me and they support me… The thing [joint pain] with Covid is basically I don’t know what I’m supposed to be looking for and nobody’s actually said anything, so I get confused sometimes when I feel ill, I don’t think of Long Covid and just think, oh maybe I’ve got a chest infection or something else. Then you go to your GP and they say Long Covid and then you don’t know what it is*.” (Female, white British, 60s, phase 2).

The findings in this theme illustrated the difficulties our LC participants faced in navigating support for their complex symptoms within the complicated secondary care system. Being “in the system” was essential for our participants access to various specialised medical resources, but the challenges persisted in supporting LC as a novel condition within an already strained healthcare system. While primary care serves as the gateway to the health system, secondary care is where patients seek improvement, if not full recovery, of their medical conditions [5, 9]. As demonstrated earlier, the lack of holistic and consistent support in specialist care could undermine the physical, emotional, social, and financial aspects of PLwLC in an ongoing manner. The slow progress in secondary care may gradually erode people’s hope to recover through receiving specialised (often as perceived “more advanced”) support. Such experiences in secondary care might have an existential dimension, as captured by Dorothy’s reflections on her vulnerability and even mortality in phase 2 interview: “*this is the final curtain, this is the last, ‘cos you think, well this isn’t getting any better, I don’t know what this is*” (Female, white British, 60 s).

### Theme 3: unique challenges to promote LC integrated care pathways

#### Still limited coverage

To provide PLwLC with more specialised and integrated care, a variety of LC clinics in England and similar services in the rest of the UK have been established. *The NHS plan for improving long COVID services* published in 2022 highlighted the accomplishments of developing new services and outlined plans to cut waiting times, improve care quality and reduce health inequalities [12]. However, our study found that these pathways were largely unknown and challenging to access for people with LC. In phase 1, only 4 out of 80 participants had accessed LC clinics through GP referrals, and this only increased to 5 in phase 2. A major contributing factor to this issue was the prevalence of barriers encountered when trying to enter and navigate healthcare systems. For example, Christine had initial difficulties accessing her GP and faced a lengthy process of medical examinations before being referred to a LC clinic (female, white British, 50 s). By her second interview, she had been on the LC clinic waiting list for over a year, despite her proactive efforts to communicate with her GP and the clinic.

The limited access also lay in a lack of awareness about LC integrated/specialised care. Approximately one third of our participants with LC in phase 1 had never heard of LC clinics or other similar services. Despite being provided with information about the integrated care pathways, many participants’ knowledge remained largely limited in phase 2. This was due to the scarcity of publicly accessible information about the specialised services and difficulties in accessing them (or the healthcare systems more generally):

Malcolm: “*I haven’t heard about a Long COVID clinic or something like that in this area if there was something I think she would have said, or if you could see, the nurse would have said, do you want to go to a clinic? But there’s nothing*.” (Male, white British, 70s, phase 2).

As seen above, inadequate information for primary care practitioners was another contributor to the limited access to LC integrated care pathways. This issue was reiterated by a GP who had to rely on her patients for information about the support offered in LC clinics:

“*I was relying on my patients, I was like, tell me what the Long COVID clinic is like and come back to me. And then I can tell the next person*”. *(GP 1, phase 1)*

The situation improved in phase 2 interview as this GP could access notes via NHS systems from the LC clinics about support details for her patients, but she still struggled to stay updated on the fast-evolving LC care pathways due to a lack of direct communication from the local LC clinics.

#### Tendency to develop a pathway towards holistic and consistent care

A rapidly evolving picture was captured across the two phases of interviews, highlighting how LC integrated pathways were developed from initially highly specialised medical care (e.g., often centred around respiratory care) towards the provision of increasingly holistic support. In phase 1, two out of the four participants who reported having access to LC integrated care pathways, found that LC clinics tended to "apply existing medical models to a new illness", be "led by respiratory specialists", and "focus on 'clinically severe and visible' symptoms" (e.g., organ damage, lung issues). One of them, Lucy, called for a “One-Stop-Shop” for more integrated and holistic care:

“*A one-stop-shop where you can, say, ‘this is Long COVID, this is what people have experienced, this is what doctors can do, this is what they can’t do, this is what people have found helpful, this is what’s available’ would be really helpful. It just seems so random, what people are getting and aren’t getting*.” (Female, White British, 50s, phase 1)

While acknowledging the heavily clinical focus of LC care pathways, practitioners also emphasised the evolving support for the holistic wellbeing of people with LC. In phase 1, a LC clinic coordinator reported the development of a comprehensive psychology-led course to empower patients to better identify their needs and manage their symptoms. Notably, there were further enhancements addressing existential needs during phase 2. A hierarchical framework of identity roles was adopted to address patients' awareness of identity loss and mortality to (re)develop a ‘flexible and resilient self’, assisting them in prioritising the recovery of their primary identity and well-being.

Improvements can also be observed in service providers' efforts to offer follow-up care that specifically addresses the fluctuating nature of LC symptoms. For instance, the previously mentioned care pathway coordinator discussed the potential for self-referral to the LC clinic. Similarly, a rehabilitation coordinator introduced their plan to implement "Patient-Initiated Follow-Ups" to empower patients to play a more active role in seeking specialised care consistently, while acknowledging their limited capacity:

“*What we’re just at the point of starting to offer are the Patient Initiated Follow-Ups, so once they’ve been through the groups that we feel have been appropriate and they’ve gone through them all once, it’s then about some sort of self-management and then offering them to see how you go on. If you feel you need to come back to us, you initiate another follow-up with us. But as I say, we’ve still got patients working through and we are not quite yet, but that’s our intention*.” (Rehabilitation coordinator 1, phase 2)

#### Uncertainties in funding security

Positive changes in LC care pathways were indeed observed over the two 2021–2022 interview rounds. However, significant uncertainty lingered regarding funding sustainability for specialised care for this persistent condition. This uncertainty was not unique to LC specialised care but reflected broader NHS resource challenges, with stretched and fragmented resources [10, 25].

Healthcare professionals in region A voiced concerns about long-term funding. Almost half of them noted how funding constraints affected daily operations, service reach, capacity, and future planning for LC specialised services. A manager for specialised medicine highlighted the persistently limited availability of funding for expanding and even maintaining the services. In phase 1 in early 2022, she underscored the challenges of prioritising funding over the LC patients’ real needs, further creating a highly uncertain future for service provision:

*“All these patients, that we’ve just talked about ending up within the community and getting referred back in, they’re not funded. We haven’t got any funding for increased activity related to Long COVID. We’ve got at the moment recovery money which means we can put on extra activity and have extra sessions for the consultants that can be funded. But long term it’s very difficult to predict how long this is going to go on for. We keep stopping and starting activity depending on spikes, so we don’t know what our backlog is going to look like and we don’t know what the long-term implications are going to be.”* (Specialised medicine manager, phase 1)

In the second interview in late 2022, these situations had not improved and, in some cases, had deteriorated. Both this manager and other service providers highlighted the potential for long-term funding insecurity to disrupt LC specialised care in a multitude of ways, affecting staff retention, consistency and coordination:

*“It’s much harder to recruit to temporary contracts and secondment, it’s destabilising for the service where you take the person from because it’s really hard for them to fill that vacancy… we are three separate Trusts working together and it’s quite complicated then because each Trust has their own policies and procedures that aren’t necessarily the same. Everyone still has a contract with one Trust and just follow those procedures from their Trust which might be different from their colleagues’ procedures. So those things are quite challenging.”* (Service manager 1, phase 2)

The funding uncertainties prompted some creative approaches to sustain LC specialised care within the constrained financial parameters. Some practitioners disclosed plans to secure additional funding. Some other managers and commissioners also deliberated the possibility of integrating LC specialised care into existing post-viral/chronic illness pathways, aiming for more consistent and potentially comprehensive care (e.g., drawing lessons from other health conditions).

To sum up, healthcare is comprised of various systems, including primary (community), secondary, and specialised care. Theoretically specialised healthcare aligns well with the philosophy of person-centred care, as both emphasise the importance of tailoring treatment and care plans to meet patients' specific and holistic needs [23]. While the specialised pathways showed some improvements towards a more holistic approach to LC, significant gaps persist. These gaps hinder the integration of LC specialised care into the healthcare system to address LC's multifaceted and persistent nature effectively. These gaps primarily result from limited access to LC specialised care, driven by funding insecurity and subsequent workforce shortages, often intertwined with issues in primary and secondary care systems. This fragmentation was exemplified by the experience of Lucy (Female, White British, 50s), who initially received physical therapy from a LC clinic but faced discontinuation and challenges accessing follow-up support within primary and secondary care systems.

## Discussion

Our study reveals persistent challenges in seeking support within the UK healthcare system for PLwLC, leading to continued barriers, delays, and disruptions in accessing treatment and understanding their complex symptoms. This extends our prior work on healthcare access issues and the extensive impact of LC on PLwLC’s wellbeing [10, 31]. It deepens our understandings of how the lingering struggles in the under-resourced and complex UK healthcare system may not only undermine PLwLC’s health needs but can also cause enduring disruptions to their identity as a holistic being.

Living with highly individualised LC conditions, our participants had to exert significant, and often repeated, efforts to access primary care (the gateway to the healthcare system). They also needed to demonstrate persistence and, at times, sheer determination in navigating the highly specialised and often inconsistent care provided through secondary care and LC specialist care pathways. While the study period between 2021–2022 revealed some practical and structural improvements (e.g., increased understanding from healthcare workers, provision of more holistic care), long-standing systemic issues, including limited access, a shortage of available treatments, and disconnections between these services, remained significant obstacles [14]. These obstacles further hindered many participants from accessing medical care for their distressing symptoms based on their desires and preferences. In essence, their fundamental needs for physical and emotional comfort as a person was not fully acknowledged and supported [22]. The insights into these multi-level issues also resonate with key actions outlined in *the Long COVID: the NHS plan for 2021/22* to expand LC health support and equity through enhanced services and care coordination within and across primary and specialist care [32].

Capturing the voices of our participants facing ongoing struggles of “being turned away” and even “being abandoned” by health services, our study highlights the vital relational and existential dimensions of healthcare in the context of LC. For many, interacting with healthcare professionals was not merely about addressing physical symptoms, but also finding an accessible and trustworthy source to make sense of their suffering and adapt to their changed lives more generally. The persistent challenges in addressing relational needs within and across the various health systems were also closely linked to their longing for meaning to justify their existence in the face of their compromised health and with a fractured body. Lack of validation from healthcare and reassurance from practitioners could elevate the risk of existential encounters with meaninglessness, particularly a profound sense of anxiety being disconnected from both their once-familiar past and their greatly uncertain future [31]. These multifaceted and deeply painful struggles affirm the *NHS plan for improving long COVID services*, aiming to enhance healthcare capacity and prioritise holistic and continuous care for PLwLC in a more interdisciplinary and inter-systematic manner [12].

Our findings also further expand on the scope of person-centredness in healthcare, moving beyond individual perspectives of dignity, choice, and autonomy to emphasise a more relational approach that situates individuals' healthcare needs within a rich matrix of relationships and socio-cultural beliefs [33, 34]. The evolving concept of person-centred care now places a greater emphasis on its holistic nature. This calls for a thorough consideration of each patient's unique experiences, acknowledging and supporting their life histories, social contexts, and the relationships that matter to them [34]. It also emphasises the importance of preserving and respecting patients’ cherished personhood, thereby preventing any unintentional harm to these esteemed facets of their existence.

This holistic perspective is particularly relevant to our study as our participants shared their experience of feeling that they had lost part, if not all, of themselves as a person to their complex and persistent symptoms [31]. Considering the extended and often unpredictable illness trajectories associated with LC, failing to provide person-centred healthcare could pose a substantial challenge to PLwLC’s ability to alleviate clinical distress and, more importantly, to find meaning in enduring suffering. Essentially, our study highlights the pressing need for person-centred care in managing chronic illnesses like LC, especially in the absence of immediate and efficient treatments. To truly support individuals in their health journeys, healthcare for LC should holistically address their multifaceted and interconnected needs, ensuring a consistent sense of identity. As such, our findings underscore the practical importance of person-centred healthcare in assisting PLwLC to gain security, understanding, and the ability to live with their health conditions as an integral part of their ongoing lives.

By examining the healthcare needs of our participants across primary, secondary, and specialised care pathways, our study enhances the holistic nature of person-centred care at a structural level. LC, along with the broader challenges posed by the COVID-19 pandemic, has exacerbated the conflicting priorities between individual care and public health interests [9]. Our findings regarding the fragmentation and inconsistency in care highlight the conceptual and empirical significance of incorporating a comprehensive approach to healthcare structures to ensure person-centred care [23]. As strongly voiced by PLwLC, their healthcare struggles often went beyond a single health system and were encountered across various provisions. The narratives shared by healthcare professionals reveal both macro systemic issues (e.g., funding supply, design and priorities of health services at different levels) and micro structural barriers (e.g., lack of inter-organisational cooperation) that impede holistic care provision to PLwLC. To better view PLwLC as a whole and living person, it is essential to bridge the gaps and inconsistencies within and across various healthcare systems. This extends beyond LC healthcare to other chronic conditions, such as chronic fatigue syndrome. Existing literature on chronic illness has highlighted empowerment as a key approach to ensure consistent holistic care for patients [35]. Building upon this, our findings suggest the significance of further integrating patients into and across different health systems for stable (e.g., funding and workforce security), continuing (e.g., consistency in care) and flexible (e.g., training and understanding) care to address PLwLC’s needs and identity through a more person centred lens.

Finally, the longitudinal focus of our study captures both the evolving healthcare needs of our participants and the importance/challenges of aligning healthcare infrastructures to address these changes. Methodologically, we generated a rich set of qualitative data spanning across 2021–22, providing insights into the fluidity of individual healthcare needs, broader health systems and also the relationships between them [28]. Our research method, designed to track changes over time, has made substantial contributions on two fronts. Conceptually, it has greatly expanded our comprehension of the evolving healthcare needs of individuals, exposing the often complex (e.g., changing or increasing) intersections between conditions like LC and other health issues. This underscores the imperative nature of adopting a holistic and person-centred approach to care, one that recognises the interconnectedness of various health dimensions, both concurrently, across different aspects of life and longitudinally over time. On a practical level, our study has underscored the critical necessity for healthcare providers to regularly review and adapt care plans to accommodate these dynamic shifts in patient needs. This highlights the vital importance of flexibility and responsiveness in healthcare strategies, particularly in chronic illness management.

## Limitations and implications

While our study offers valuable insights into the challenges and complexities of person-centred healthcare for LC, there are several limitations to consider. Firstly, limited information was reported by our participants on LC community-based care, such as physiotherapy and rehabilitation services, as well as tertiary care. These areas require further exploration to comprehensively address the diverse healthcare needs of LC suffers. Secondly, our study's focus on healthcare professionals in region A may not fully represent the healthcare experiences and developments in other geographic locations, warranting future research in more diverse settings. Additionally, whilst we engaged with a socio-economically and ethnically diverse sample, the absence of Black minority participants in our study highlights the further need for research that captures the unique healthcare experiences within this group and beyond. Lastly, our study generated rich data, underscoring the importance of future research to further explore the multifaceted challenges and opportunities in person-centred care for LC and to potentially extend these findings to inform care for other chronic conditions.

### Supplementary Information

**Supplementary Material 1.**

**Supplementary Material 2.**

**Supplementary Material 3.**

**Supplementary Material 4.**

## Data Availability

The data that support the findings of this study are available on request from the corresponding author. The data will be submitted to the UK Data Service following the completion of the CONVALESCENCE study.

## Abbreviations

GP: General Practitioner
LC: Long COVID
NHS: National Health Service
ONS: Office for National Statistics
PLwLC: People Living with Long COVID
UK: United Kingdom
