# Correction: “They seemed to be like cogs working in different directions”: a longitudinal qualitative study on long COVID healthcare services in the United Kingdom from a person-centred lens

**DOI:** 10.1186/s12913-024-11015-x

**Published:** 2024-04-25

**Authors:** Chao Fang, Sarah Akhtar Baz, Laura Sheard, J. D. Carpentieri

**Affiliations:** 1 Department of Sociology, Social Policy and Criminology, University of Liverpool, Liverpool, UK; 2 Department of Health Sciences, University of York, York, UK; 3 Department of Education, Practice and Society, Institute of Education, University College London, London, UK

**Correction to**: ***BMC Health Services Research*(2024) 24:406**.

10.1186/s12913-024-10891-7.

In this article, some of the reference citations were not formatted as reference citations. The first citation to each of the references was formatted correctly, but every repeated citation for each of the references was formatted as plain text. These errors were caused as the result of a typesetting mistake.

The original article has been updated to correct the formatting for the affected reference citations. The publisher apologises to the authors and readers for the inconvenience caused by this error.

