# Supplementary material for: “They seemed to be like cogs working in different directions”: a longitudinal qualitative study on Long COVID healthcare services in the United Kingdom from a person-centred lens
Source: BMC Health Serv Res. 2024 Apr 1;24:406. doi: 10.1186/s12913-024-10891-7 (PMC10986002; doi:10.1186/s12913-024-10891-7)
Supplement: Supplementary file 1 — Supplementary Material 1. [file 12913_2024_10891_MOESM1_ESM.docx]

**Phase 1 Topic Guide: people living with Long COVID**

**1. Introduction**

We are interested in talking to you about your experience of living with symptoms of Covid. We know from the Born in Bradford survey/UCL cohort study survey you stated you had Covid symptoms for more than four/five/twelve weeks. Thanks very much for answering questions about Covid on the survey. Today, I'd like to have a more in-depth conversation about your experiences of living with Covid.

We are aware that some people experience longer Covid symptoms than others. You may have heard of the phrase “Long Covid'' as it is an increasingly popular term (although we are aware that not everyone with Covid symptoms for more than a month would identify as having ‘Long Covid’). We are carrying out this study to understand more about the experiences of living with Covid symptoms for about 5 to 12 weeks or more. We aim to provide evidence to improve practice and policy.

This interview will explore the impact that Covid has on your everyday life and your experiences of accessing health care support for Covid symptoms. The interview will last between no more than 1 hour. Importantly, you do not have to answer any questions you are not comfortable with. You can also stop or pause the interview at any time. You have the right to withdraw during and after the interview - any data collected will be destroyed if you decide to withdraw. If you would like me to repeat any question or provide further explanation, please feel free to ask. You can also ask questions at any time during the interview.

**2. Opening question/ice breaker**

- Tell me a bit about yourself (e.g. are you working/studying/retired)

**2.1 Initial experience of Covid**

- Can you remember when you first had Covid-19 symptoms?
- Tell me about your initial Covid-19 experiences.
- Did you get a test? (lateral flow or PCR?) What happened?
- Did you have any contact with your GP, the hospital or other healthcare services? Can you tell me more about that?
- What support did you seek when you got Covid-19?

**2.2 Thoughts about Long Covid and experiences of prolonged symptoms**

- What do you think about the term “Long Covid” and do you think it applies to you? If not, why not and what would be a better term?

[From this point onwards, use the patient’s self-defined term to describe their Covid symptoms]

- Do you experience any Covid related symptoms now?
- How long do you think your Covid symptoms have lasted?
- Would you say your health was good before getting Covid? If not, what conditions did you have?

**3. Managing the illness**

**3.1 Impact on day-to-day life**

- How have the Covid symptoms affected your life?
- What changes have you noticed - describe how your daily routine has changed compared to your days prior to having Covid (e.g., could you give me an example of a typical (week)day before getting Covid and then a typical day after getting it?) [Dyad question]
- How did you try to maintain your normal routine? Did you find it was challenging?

**3.2 Managing symptoms**

- Tell me about the symptoms you have experienced over time. Did the Covid symptoms change?
- What sort of things have you been doing to help manage/cope with your symptoms?
- Have you got any strategies to manage the symptoms? If yes, what are the strategies?
- How have your physical activities been affected by your Covid symptoms? What changes have you noticed? (e.g., is there an activity you are no longer able to do / you find more challenging now compared to pre-Covid?)

**3.3 Impact on mental health**

- Has your Covid experience impacted your well being in any way? (feeling frustrated, stressed, sad, angry)
- How are you feeling now regarding your experience of having to live and cope with Covid?

**4. Health care services**

**4.1** **Experiences with GPs/hospitals/health services**

- Have you asked for medical advice, support or treatment for Long Covid?
- Did you contact your GP? What happened? Who did they refer you to?
- What advice were you given and was it useful?

**4.2 Barriers and levers to access**

- Have you faced any barriers or difficulties when accessing healthcare? If so, what are they?
- Can you tell me about anything that was good about accessing healthcare support that helped you.

**4.3 Support required/interventions**

- What further healthcare support do you think would help with your recovery?
- Reflecting on your experiences, what improvements do you think are needed to better support people with similar experiences as you? Why do you think so?

**4.4 Vaccination (optional)** - If participants do not wish to discuss this, we will skip the questions below.

- If you don’t mind, can you tell me if you have received the Covid vaccine? If so, how many doses have you received?
- Do you think vaccines have helped you recover from your symptoms? If so, how?

**5. Role of family and friends**

**5.1 Impact on family life/relationships**

- Tell me a bit about your family or those living with you.
- Did you seek and/or receive support from your family or friends? If so, what support?
- How has your illness impacted them and your relationship with them [This may not be asked in a dyad interview]

**5.2 Responsibilities**

- How have your Covid symptoms impacted your ability to [e.g. participate in family life, volunteer, work]?
- What changes or difficulties have you and your family members experienced? [e.g., increased caregiving burden, changing family roles].
- Do you have any caring responsibilities in your family/social circle? (e.g., childcare, caring for an ageing/sick family member/friend).
- How have your Covid symptoms affected your ability to provide care?
- How have the changes in caring responsibilities altered your relationships/roles in your family? How did you cope with these changes/difficulties?

For dyad family members/friends

- How important has the support from your partner/family member/friend been while having Long Covid? What aspects have you both struggled with?
- How did your family/friend’s Covid experiences impact your life and/or the whole household?

**6. Online support groups**

- Have you accessed support from any Long Covid support groups, either face-to-face or online (e.g., Twitter, Facebook group)?
- What is your experience of engaging with these groups?
- How has this shaped your experience of living with Long Covid?

**7. Socio-economic impact**

**7.1** **Impact on employment**

- Do you work? (Yes - What job do you do? No - Did you work before having Covid?)
- How has Long Covid impacted your ability to work?
- Do you think your employer was helpful in terms of supporting you during your Covid illness? If so/or not so, could you expand your answer further?
- Have you experienced any barriers when returning back to your normal work routine? If any, can you further explain your answer to me?
- What support did/do you require to get back into work?

**7.2 Financial impact and benefits (prompt answering is optional)**

- Have you experienced changes in your finances after getting Covid? (and reducing work hours/losing job/receiving benefits)
- What are your experiences of leaving/reducing employment due to Covid? What support did you receive from your family, communities and/or the Government?
- There have been discussions about including patients with prolonged Covid experiences into the disability benefits (e.g., using this as an eligibility criterion for certain types of benefits). What do you think?

For dyad family members/friends

- How has the impact of Covid on your partner’s employment/finances impacted the household?

**8. Impact on identity**

- Has Long Covid changed who you are? (identity as parent or worker etc.)
- Thinking about your future: First, what are your hopes, in terms of Covid and your health, and any of the impacts you've talked about today?
- Do you have any fears? What are they?
- If you could receive more support in the future regarding your Covid experiences, what kind of support would you like to receive and from where/whom?
- (For recovered participants) What support would you like ongoing Long Covid sufferers to receive?

For dyad family members/friends

- Do you think Long Covid has changed your family/friend in terms of [e.g., used to be active and sporty, someone with confidence, breadwinner for the family]?

**9. Finishing off**

- Is there anything else you would like to add? Anything we haven’t covered or you want to discuss more about?
- Do you have any questions?

**10. Debrief:** Thank you for taking part in this interview. Please feel free to email or phone me if you have any further questions. Over the course of the project we will inform you about outputs from the project. I will also be in touch around May 2022 to arrange the second interview - I will be in contact closer to the time.
