# Supplementary material for: “They seemed to be like cogs working in different directions”: a longitudinal qualitative study on Long COVID healthcare services in the United Kingdom from a person-centred lens
Source: BMC Health Serv Res. 2024 Apr 1;24:406. doi: 10.1186/s12913-024-10891-7 (PMC10986002; doi:10.1186/s12913-024-10891-7)
Supplement: Supplementary file 2 — Supplementary Material 2. [file 12913_2024_10891_MOESM2_ESM.docx]

**Phase 1 Topic Guide: healthcare and public health professionals**

**Introduction**

We are interested in talking to you about your professional experiences providing health services and support to Long Covid patients. We are aware some people experience longer Covid symptoms than others, this is increasingly termed as Long Covid (amongst the public and medical professionals). We are carrying out this study to understand more about people’s experiences of living with Covid symptoms for about 5 to 12 weeks or more, as well as their experiences of accessing healthcare. We are also exploring the experiences of healthcare professionals. Overall, we aim to provide evidence to improve healthcare services and policy to better support LC patients.

Today, I’d like to have an in depth conversation about your experiences of supporting people with LC. The interview will last between no more than 1 hour. Importantly, you do not have to answer any questions you are not comfortable with. You can also stop or pause the interview at any time. You have the right to withdraw during and after the interview - any data collected will be destroyed if you decide to withdraw. If you would like me to repeat any question or provide further explanation, please feel free to ask. You can also ask questions at any time during the interview.

1. **Opening questions**

1.1 What is your job and specific specialism?

1.2 What does this role involve?

1.3 When did you initially come across Long Covid? What happened? Do you think it is a completely new illness or similar to something you have come across before?

1.4 Reflecting on your own understanding and encounters with patients, how would you describe or define ‘Long Covid’?

1. **The health context of Bradford**

2.1 Could you describe the health issues faced by people in Bradford that you encounter in your daily practice.

2.2 Has Covid and particularly Long Covid exacerbated these issues and health inequalities? If so, how?

1. **Supporting Long Covid Patients**

3.1 How are Long Covid patients referred to you?

3.2 In what ways do you support patients with Long Covid?

3.3 What health issues or symptoms do patients come with? (e.g. physical, cognitive, mental health)

3.4 What advice do you provide for Long Covid sufferers?

3.5 What services do you refer them to? Have you referred patients to Long Covid clinics or any rehabilitation services? How will this support them with recovery?

3.6 Overtime, what have you learnt about Long Covid and how to better support patients?

3.7 In our interviews with LC patients, some reported inconsistency in care and support when trying to access healthcare, especially when trying to seek support across multiple organisations. Have you heard or experienced anything like this?

3.8 In addition to medical concerns, have you come across any other challenges faced by your LC patients (e.g., social, familial, financial).

1. **Long Covid at the intersections**

4.1 Do you find some people require more specific support than others for Long Covid or have more complex symptoms? (for example, elderly/middle age/young, women/men, Black and ethnic minorities, those with underlying health conditions).

4.2 Reflecting on your practice, do any underlying health conditions further exacerbate people’s experiences of Long Covid?

1. **Improvements**

5.1 Have there been any improvements in services over time? What improvements have been made? If not, why do you think so?

5.2 What has worked well for Long Covid patients?

5.3 What challenges have you faced when supporting/navigating your LC patients?

5.4 What improvements do you think are required to better support Long Covid patients? (both in your practice, the wider healthcare services and policy).

5.6 Beyond health care, what other forms of support do you think Long Covid patients require to receive more holistic care? (e.g. from employers, financial, mental health services, rehabilitation).

1. **Finishing off**

6.1 Is there anything you would like to add, anything we have not covered.

6.2 Do you have any questions?

**Debrief**

Thank you for taking part in this interview. Please feel free to email or phone me if you have any further questions. Over the course of the project we will inform you about outputs from the project. I will also be in touch around May 2022 to arrange the second interview - I will be in contact closer to the time.
