# Supplementary material for: “They seemed to be like cogs working in different directions”: a longitudinal qualitative study on Long COVID healthcare services in the United Kingdom from a person-centred lens
Source: BMC Health Serv Res. 2024 Apr 1;24:406. doi: 10.1186/s12913-024-10891-7 (PMC10986002; doi:10.1186/s12913-024-10891-7)
Supplement: Supplementary file 3 — Supplementary Material 3. [file 12913_2024_10891_MOESM3_ESM.docx]

**Phase 2 Topic Guide: healthcare and public health professionals**

**Tailor to participant reflecting on first interview**

This topic guide will use broad and open prompt questions which allow the interview to be participant led. Questioning will need to be adapted depending on the work history of participants and their W1 interview. The below guide has been written for people who have provided care and support to people with LC in Bradford. Questioning may be different for those who only encounter long Covid patients occasionally or peripherally or for those in other clinical settings.

**Introduction**

Thank you for taking part in the first interview for the LC study. Today we are interested in follow-up on our first interview on the progress of services for LC and your ongoing experiences of supporting and caring for people with LC. We are also re-interviewing people with LC to understand their ongoing experiences and use of healthcare services.

A key and striking finding from our first set of interviews were the barriers people faced in accessing healthcare support for LC in Bradford - we have been able to share these findings to healthcare professionals and practitioners, for example in NHS England regional meetings. We aim to continue this dialogue and explore changes overtime.

Today, I’d like to have an in depth conversation about your ongoing experiences of supporting people with LC. The interview will last no more than 45 mins (unless you would like to talk for longer). Importantly, you do not have to answer any questions you are not comfortable with. You can also stop or pause the interview at any time. You have the right to withdraw during and after the interview - any data collected will be destroyed if you decide to withdraw. If you would like me to repeat any question or provide further explanation, please feel free to ask. You can also ask questions at any time during the interview.

**Questions**

**1, Introduction service questions**

- Since the last time I spoke to you, are you in the same role for supporting LC patients?
  - If not, what is your role now? Why did you change roles?
- How has your particular service developed [since the last time I spoke to you at X month]?
  - What improvements have been made? What challenges have you faced [in making these improvements]? Why did you decide to make this change?
  - Have you received any training for supporting people with LC? [in first interview many were learning as they go]. Tell me more about this. In what ways has this helped you?
- Can you describe your average working day as a clinician/manager at the LC clinic [or as a … supporting LC patients].
- What are the main persisting symptoms LC patients are still coming to you for?
- Can you describe how patients are now referred to the LC clinic?
- What support, care or treatment do you provide them with?
- What challenges are your patients facing?
- Do you provide care to LC patients in other clinical settings? If so, how do these other settings contrast with the LC clinic?

*Let the participant talk until they have exhausted their narrative. Note down anything of interest that is not covered by the above whilst they are talking and return to it to prompt for more narrative, where appropriate.*

**3. Challenges**

- What are the main challenges of working in your role?
- What patient access issues to support for LC OR the LC clinic are you aware of?
- Are there any patient groups which you still believe LC services are not reaching or are underserved and face more complex challenges?
- How can these barriers to access for these groups be addressed?
- What specific broader problems in the NHS are also impacting LC services? [e.g. understaffing, backlogs, bureaucratic issues, fragmented services)

In our first interviews, lack of access or difficulties in accessing GPs was a common finding. GPs are often the first point of contact for patients, provide an initial assessment and refer patients to the LC clinic service.

- Given this barrier in access, what other routes to accessing LC services do you think should be made available?
- We also found that there is a long waiting list to access the LC service, can you tell me more about this and the challenges it has caused?

Some patients have also reported inconsistency in care and support when trying to access healthcare, especially when trying to seek support across multiple services.

- Have you heard or experienced anything like this?
- How do you think services can be improved?

In our interviews with LC patients outside Bradford, we found that there are significant regional disparity in terms of LC specialist care/LC clinics.

- Are you aware of any regional/geographical differences in relation to accessing LC care and support?
- The LC clinic in Bradford is a good example of developing services to provide holistic support for LC patients. What advice would you like to give to your colleagues in NHS/healthcare to further improve care and support for LC patients?
- OR reflecting on the Bradford LC clinic, if you were advising colleagues on setting up a new LC clinic what would be the essential components or services that you would recommend them to include?

**4. Other questions**

- In addition to medical concerns, what are the other challenges your patients have continued to face (e.g., social, familial, financial, sick leave). Tell me more about these.
- What are the main satisfactions of working in your role?
- How has working in LC services and supporting people with LC affected you personally?
- Do you think there is enough awareness of what LC is (amongst your patients, HCPs wider public)?
- Do you think that LC should be classed as a disability?
- Reflecting on your experience, how would you define LC?
  - Any changes in the way you define it? e.g. duration, symptoms, other complexities.

**5. Questions specifically for public health/third sector workers interviewees**

- Can you describe to me the continuing work you do surrounding LC and LC services in Bradford/regionally. E.g. around physical activity.
- How are you [Bradford city council public health team/voluntary sector] continuing to be involved in (developing) LC services?
- [If running a specific community service] Can you describe how participants are referred to your service.
- In what ways has your service been continuing to support those with LC?
- In what ways are you engaging with healthcare service providers?
  - What challenges are you facing?
  - What opportunities does this provide in supporting people with LC?
- What patient access issues to LC services are you aware of? OR - You discussed X patient access issue in our last interview, is this issue still occuring?
  - How have you tried to address this issue?
  - What challenges have you faced?
- How are you and your services continuing trying to reach out to those from diverse backgrounds, such as ethic minorities, different age groups, homeless, etc.
  - How can they be supported better?
- What wider community services are available for those with LC?
  - How can these be improved?
  - Are you aware of whether they can refer people to the LC clinic?
- [Senior management question] From a managerial/structural perspective, what challenges and barriers do you think your LC services are facing or may face in the future?

**6. Wrapping up**

Is there anything you would like to add which I may have missed off?

Do you have any questions?
