# Supplementary material for: “They seemed to be like cogs working in different directions”: a longitudinal qualitative study on Long COVID healthcare services in the United Kingdom from a person-centred lens
Source: BMC Health Serv Res. 2024 Apr 1;24:406. doi: 10.1186/s12913-024-10891-7 (PMC10986002; doi:10.1186/s12913-024-10891-7)
Supplement: Supplementary file 4 — Supplementary Material 4. [file 12913_2024_10891_MOESM4_ESM.docx]

**Phase 2 Topic Guide: people living with Long COVID**

Thank you very much for taking part in the first interview and agreeing to further discuss your Long Covid and wider life experiences in this second interview. Today, I would like to catch up on your experiences of having Long Covid since the last time we spoke. In our first round of interviews, many people mentioned that they were able to draw upon previous challenges they faced in their life to help deal with their Long Covid challenges. That is why today we would also like to learn more about your life history and past experiences.

The wider findings based on the first set of interviews have been presented at various meetings with different actors: e.g., NHS England, Bradford City Council and the Government. We continue to aim to further inform policy and practice.

The interview will last between 50 minutes to 2 hours, unless you would like to speak for longer. Importantly, you do not have to answer any questions you are not comfortable with. You can also stop or pause the interview at any time. You have the right to withdraw during and after the interview - any data collected will be destroyed if you decide to do so. If you would like me to repeat any question or provide further explanation, please feel free to ask. You can also ask questions at any time during the interview.

**Introduction**

**Part 1: Review of first interview**

During the first interview we discussed ….. [tailor to participant]. Today I would like to begin with learning more about your Long Covid illness, what has changed overtime and what has happened since the last time I spoke to you which was around six months ago in XXXX (month and year). I will then ask more broader questions about your life history.

**Part 2: Update on Long Covid**

**2.1 Continued experiences of symptoms**

- Do you still experience any Covid related symptoms now?
- How have your Long Covid symptoms developed overtime, have you seen any changes, improvements or have symptoms worsened?
- [If recovered] Tell me more about your journey to recovery? What happened? What helped?

**2.2 Impact on day-to-day life**

- How have the symptoms continued to affect your everyday life?
- In what ways has your normal routine changed?
- What have you continued to find challenging?

**2.3 Managing symptoms**

- What sort of things have you been doing to help manage/cope with your symptoms?
- Have you got any strategies to manage the symptoms? If yes, what are the strategies?
- How have your physical activities continued to be affected by your symptoms? What changes have happened overtime? (e.g. have you gradually started to engage in physical activity/what challenges have you faced).

**2.4 Impact on mental health**

- Has your Covid experience continued to impact your well-being in any way? (feeling frustrated, stressed, sad, angry)
- How are you feeling now regarding your experience of having to live and cope with Covid?

**2.5 Healthcare services**

- Follow up on any specific healthcare support issues raised in the first interview e.g. did the participant get an appointment with the neurology service which they have been on the waiting list for, what happened? Did they go for private healthcare?
- Have you used any healthcare services since the last time I spoke to you?
- Have you since contacted your GP? What happened? Who did they refer you to?
- What advice have you received? Has this been useful? If not, why not?
- Have you continued to face any barriers in accessing healthcare support?
- Can you tell me about anything that was good when accessing support that helped you?
- Have you gained access to the Long Covid clinic? Tell me more about this experience.
- What further healthcare support do you think will help with recovery?
- Reflecting on your recent experiences, what improvements do you think are needed to better support you and others with similar experiences?

**2.6 Family life and support networks**

- How has your illness continued to impact your family or friends and your relationship with them?
- How have your Covid symptoms impacted your ability to [e.g. participate in family life, volunteer, work]? Have there been any changes from the last time we spoke?
- What changes or difficulties have you and your family members continued to experience? [e.g., increased caregiving burden, changing family roles].
- Do you have any caring responsibilities in your family/social circle? (e.g., childcare, caring for an ageing/sick family member/friend). How have your Covid symptoms affected your ability to provide care?
- How have the changes in caring responsibilities altered your relationships/roles in your family? How did you cope with these changes/difficulties overtime?

**2.7 Continued socio-economic impact**

***Employment***

- How has Long Covid impacted your ability to work?
- [If they were not working W1] Have you returned back to work [or education]? If not, why not?
- Have you experienced any barriers when returning back to your normal work routine? If any, can you further explain your answer to me?
- [those that have continued to work] What support did/do you require at work to help manage your illness?
- What improvements can be made by employers to further support you?

***Financial impact (optional)***

- From the last time I spoke to you, have you experienced any changes in your finances?
- If you have reduced work due to Covid, what financial support have you received?
- What improvements can be made to further support you financially?

**2.8 General Long Covid opinion questions**

- Do you think there is enough awareness of what Long Covid is (in the public, healthcare workers, workplace, amongst your friends and family)?
- Recently, it has been decided that Long Covid is not classed as a disability (although this was successfully challenged in a tribunal). What is your opinion about this?

**2.9 Impact on identity and future**

- Last time, you mentioned that you feel the sense of who you are/ your identity [was not] OR [is] challenged by Long Covid. Are there any changes in that regard? (impacted identity as a parent or worker etc.)
- As you continue to recover or have recovered, what are your hopes for the future and your health? What are your fears?
- If you could receive more support for Long Covid in the future what kind of support would you like to receive and from where?

**Part 3: broader life history**

In this last section I would like to ask you about your life history more generally, because we are interested in knowing how events/challenges you faced in earlier life has shaped your experiences in ongoing lives. For example, in the first set of interviews many people mentioned other health conditions or history of accessing healthcare and how they drew on these experiences today.

Opening question (open broad question)

**3.1** Could you please me a bit about your lives, for example, where you live/migration story, your family, your work/study.

- Have you always lived in [Bradford/ X City]? [No - where else have you lived? How long have you lived in your current city of residence?]
- What are your experiences of growing up in X?
- What challenges have you faced?
- What have you liked about living in X? What do you not like?

**3.2** Those who migrated to the UK:

- What was your life like living in X country?
- When did you come to the UK? Who else came with you?
- What was your experience of leaving your home country and coming here?
- What was your experience of settling in the UK/X city? E.g. what was your experience of interacting with people, were people welcoming?
- What challenges did you face?
- What opportunities did this provide?
- How are you feeling now about living in X city?
- In the first interview, you compared health services in the UK to your home country. Could you tell me more about this, what differences are there, drawing on your experience are healthcare services better here or there?

**3.3** Could you tell me about any significant challenges (other than the health issues you have mentioned earlier) you have faced in your life? (e.g. - these could be challenges in your personal life, school, working life or health).

- Please tell me more about this.
- What happened?
- How did this impact your everyday life?
- How did you address or manage this challenge?
- Did you receive any support from your family, friends or any professionals?
- Did this impact your personal relationships? In what ways? How did you cope with these changes/difficulties overtime?
- What did you learn from this experience and how does it impact your life today? (negative impacts, positive impacts, lessons learnt, does it help you deal with other challenges in your life)
- In what ways has this shaped you as a person?

Have there been any positive events in your life that you draw upon/look back to today?

- What happened?
- How has this impacted your life?.... [above questions]

**3.4 Experiences of loss and grief**

[only for interviewers, both research and our own data have suggested that LC patients may experience a sense of grief due to multifaceted losses caused by or connected to living with LC. This section, thus, is designed to capture participants’ general experiences of loss and grief as these date? will be useful to better understand what loss means to LC patients and how they feel and deal with the voids of meaning and identity in their lives.]

- Have you lost anyone close in your life (skip this if they have talked about this in the previous section).
- How did you feel and how did you deal with your loss and grief.
- Some people said living with LC can give rise to a sense of grief, have you experienced anything similar? If so, are there any differences between your LC-related grief and your grief regarding your loved one?

*Those in old age*

- In a sense, everyone is ageing. I wondered if you have thought about your experiences of getting older and the implications on your lives.
- Have you experienced any particular challenges in relation to your age and ageing?
- Have you ever felt loneliness or disconnected from others?
- Do you think you have enough people to talk about your feelings and concerns? Do you like to confide in other people about your emotions.
- When reflecting on your lives, do you have any regrets or prides? If so, can you tell me a bit more about these and if these still impact your lives.

**3.5 Plans for the future**

- Has Long Covid or the pandemic impacted your plans?
- Do you have any plans for your future, e.g. education, career, family life?
  - For those youngers: education, career planning
  - For those mid-age: career, family-planning and possibly retirement and ageing.
  - For older participants: plan for their ageing and dying (this can be sensitive, so we can decide whether to ask about dying depending on the conversation flow).
- What are your hopes for the future? Do you have any fears?
- Are there any uncertainties ahead of your life? If so, what are these uncertainties?

**3.6 Previous health illnesses**

- What major illness or injuries have you had in the past? Or You mentioned you had [tailor to participant e.g., arthritis, diabetes) - could you tell me more about this?
- How did this impact your everyday life?
- How did you manage it?
- Do you think the experience of managing this illness helped you deal with your Long Covid symptoms? Have there been similarities in your experiences? Do you use any similar strategies to manage Long Covid symptoms?
- Could you please tell me more about your experiences of having both X illness and Long Covid? What difficulties did you face?

**3.7 Previous engagement with healthcare services**

- How have your previous [positive or negative] encounters with healthcare services [e.g. you mentioned encounters of racism or the GP not believing you] shaped your experiences today of accessing healthcare?
- Could you tell me more about why you prefer not to use healthcare services or medicine when you are ill? What other strategies do you use to manage illnesses?
- Has having Long Covid changed your views on the use of medicine and healthcare support? If yes, in what ways? If not, why not?

**3.8. Covid pandemic experience and end to restrictions (this can be used as optional questions when the interviewer runs out all above questions)**

- Could you tell me about your experiences during the Covid-19 pandemic lockdowns. For example, how did the lockdowns and other restrictions impact your everyday life and wellbeing?
- Now that Covid restrictions have been lifted, what changes have occurred in your everyday life? (prompts: family life, work, accessing healthcare, wellbeing)
- What precautions do you continue to take?
- What challenges are you facing?
